# Post-Amputation Pain Is Associated with the Recall of an Impaired Body Representation in Dreams—Results from a Nation-Wide Survey on Limb Amputees

**DOI:** 10.1371/journal.pone.0119552

**Published:** 2015-03-05

**Authors:** Robin Bekrater-Bodmann, Michael Schredl, Martin Diers, Iris Reinhard, Jens Foell, Jörg Trojan, Xaver Fuchs, Herta Flor

**Affiliations:** 1 Department of Cognitive and Clinical Neuroscience, Central Institute of Mental Health, Medical Faculty Mannheim, Heidelberg University, Mannheim, Germany; 2 Sleep Laboratory, Central Institute of Mental Health, Medical Faculty Mannheim, Heidelberg University, Mannheim, Germany; 3 Division of Biostatistics, Central Institute of Mental Health, Medical Faculty Mannheim, Heidelberg University, Mannheim, Germany; 4 Department of Psychology, Florida State University, Tallahassee, Florida, United States of America; 5 Department of Psychology, University of Koblenz-Landau, Landau, Germany; Birkbeck, University of London, UNITED KINGDOM

## Abstract

The experience of post-amputation pain such as phantom limb pain (PLP) and residual limb pain (RLP), is a common consequence of limb amputation, and its presence has negative effects on a person’s well-being. The continuity hypothesis of dreams suggests that the presence of such aversive experiences in the waking state should be reflected in dream content, with the recalled body representation reflecting a cognitive proxy of negative impact. In the present study, we epidemiologically assessed the presence of post-amputation pain and other amputation-related information as well as recalled body representation in dreams in a sample of 3,234 unilateral limb amputees. Data on the site and time of amputation, residual limb length, prosthesis use, lifetime prevalence of mental disorders, presence of post-amputation pain, and presence of non-painful phantom phenomena were included in logistic regression analyses using recalled body representation in dreams (impaired, intact, no memory) as dependent variable. The effects of age, sex, and frequency of dream recall were controlled for. About 22% of the subjects indicated that they were not able to remember their body representation in dreams, another 24% of the amputees recalled themselves as always intact, and only a minority of less than 3% recalled themselves as always impaired. Almost 35% of the amputees dreamed of themselves in a mixed fashion. We found that lower-limb amputation as well as the presence of PLP and RLP was positively associated with the recall of an impaired body representation in dreams. The presence of non-painful phantom phenomena, however, had no influence. These results complement previous findings and indicate complex interactions of physical body appearance and mental body representation, probably modulated by distress in the waking state. The findings are discussed against the background of alterations in cognitive processes after amputation and hypotheses suggesting an innate body model.

## Introduction

The amputation of a limb represents the most serious breach of one’s body integrity. The majority of limb amputees report awareness of a phantom limb [1], [2], i.e., the perceived presence of the missing limb. Beside the mere awareness of a phantom limb, amputees report various kinds of non-painful phantom sensations, such as tingling sensations or thermal perceptions [1], [2], or telescopic distortions, i.e., the sensation that the phantom limb has changed its length, most often shortened, over time [3]. Many amputees complain of phantom limb pain (PLP), which describes a painful sensation perceived in the removed limb. Mean PLP rates of about 75% have been reported in epidemiological studies [1], [2], [4], [5], [6], [7], [8], [9], [10]. Additionally, about 61% of the limb amputees reported residual limb pain (RLP), i.e., pain in the still present portion of the limb close to the amputation line [1], [2], [4], [5], [11]. In combination, PLP and RLP account for a significant reduction of health-related quality of life [12], highlighting the clinical importance of post-amputation pain.

In non-amputated persons, the subjective level of well-being is associated with dream content. Pesant and Zadra [13] showed that negative affect during the waking state is related to dream content categorized as aggression, failure and misfortunes, or a general negative emotional bias. These results support the so-called continuity hypothesis, stating that dream content relies basically on experiences in the waking state [14]. Assuming that amputees draw on everyday experiences in their dreams, aversive amputation-related sensations might also be present during dreaming. This should also be true for the ever-present impairment of the physical body as well as the accompanying sensory experiences after amputation.

There are only a few studies mentioning dreamed body representation in amputees to date (for a review see [15]), and up to now, there is only one study with an epidemiological approach to investigate this issue in amputees [16]. This study found that 31% of 146 participants, who were able to remember dream content, recalled themselves as mostly or always intact (i.e., as before amputation). A minority of 21% of the amputees recalled their body as mostly or always impaired (i.e., as after amputation). Another 37% of the amputees reported that they dreamed of their bodies in a mixed fashion, i.e. sometimes as intact, and sometimes as impaired. Due to the high number of amputees who recalled themselves as having an intact body representation in dreams, the authors assumed the existence of an innate body model, predominantly opposing physical changes. However, the study by Mulder et al. [16] excluded amputees without dream recall, and did not take dream recall frequency into account. This appears to be problematic since subjects with a low dream recall frequency have difficulties to give estimates about their dream content [17]. Furthermore, most studies mentioning the amputee’s body representation in dreams [15] used rather coarse measures for the assessment of body representation, probably biasing the results.

As far as we know, neither the study by Mulder et al. [16] nor any other study investigated whether amputation-related sensations such as post-amputation pain in the waking state affect the body representation in dreams. The continuity hypothesis [18] would predict that each factor which increases the salience of the waking life experience (here: the impaired body integrity in waking life), should result in a higher percentage of dreams with impaired body representation. Thus, the dream content in amputees might match the patient’s psychological and physical state.

In the present study, we examined the relationship between the presence of post-amputation pain, other amputation-related features, and the recall of body representation in dreams in a large nation-wide survey of German amputees in the context of the PHANTOMMIND project. We hypothesized that there is a positive association between the presence of post-amputation pain and the recall of an impaired body representation in dreams.

## Methods

### Study Design and Sample Recruitment

Persons suffering from an amputation performed on their upper or lower limbs were surveyed using a questionnaire assessing amputation-related information and phantom sensations, based on the Phantom and Stump Phenomena Interview [19], complemented by items assessing dream recall frequency [20] and recalled body representation in dreams. They were recruited through state agencies so that their anonymity was preserved until they contacted the project and decided to participate in the study. All participants gave written informed consent prior to the procedure, and the study and consent protocols adhered to the Declaration of Helsinki and were approved by the Medical Ethics Commission II of the Medical Faculty Mannheim, Heidelberg University.

### Research instrument

The questionnaire consisted of a total of 53 items. In a general part, we assessed demographic data of the participants such as age and sex. In a clinical part, we assessed self-reported lifetime prevalence of mental disorders as well as the presence of sleep disorders. Of special importance in the context of the present study were items aiming at amputation characteristics, post-amputation experiences for the affected limb, and amputation-related dream content.

**Amputation characteristics**. The participants were asked whether they suffered from congenital abnormalities in limb development or whether they suffered from a limb amputation after birth. They were further asked to indicate the affected limb (upper or lower limb), the site of amputation (right- or left-sided amputation), and the level of amputation on the basis of a body drawing, which was further used to calculate the percentage of residual limb length. Furthermore, they were asked when their amputation was performed (year and month). Together with the date of return, we calculated the time since amputation (in years). Additionally, participants had to indicate whether or not they used a prosthetic device (regardless of prosthesis type) to compensate limb loss.

**Post-amputation experiences**. The participants were asked whether they had experienced PLP and RLP in the last three months. Both pain types were assessed separately and introduced by a short description of the pain in order to clarify the difference between PLP and RLP. The response alternatives were a) *No*, *I have never experienced PLP/RLP*, b) *No*, *I do not experience PLP/RLP currently*, *but I did so in the past*, and c) *Yes*, *I currently experience PLP/RLP*. The first two response alternatives were pooled as current *absence of PLP/RLP*, whereas the last response alternative was coded as current *presence of PLP/RLP*. Additionally, we asked for the general presence of non-painful phantom sensations using the same categories as used for the post-amputation pain experiences mentioned above. Specifically, the participants were further asked for the presence of a telescope, i.e., the perception of a shortened phantom limb, which also appears to represent a dysfunctional, non-painful adaptation after limb amputation [21].

**Dream questions**. Dream recall frequency was recorded on a seven-point scale (coded as 0 = never, 1 = less than once a month, 2 = about once a month, 3 = two or three times a month, 4 = about once a week, 5 = several times a week, and 6 = almost every morning).

Participants who responded affirmatively in the dream recall frequency scale (response > 0) were asked to indicate the distribution of recalled body representation in dreams on a percentage basis: “How are your dreams distributed among the following categories? Your indications should add to 100%”. The three response categories were a) impairment due to amputation is present in the dream, b) the body in the dream is intact and no impairment occurred, and c) perception of the own body in dreams could not be remembered. These data were only entered into statistical analyses when the added percentage indications had a deviation of a maximum of 1% from 100%, reflecting minor rounding errors, since otherwise the indications were implausible and suspected to be invalid. Data of n = 51 amputees did not meet this criterion.

### Sample

In total, 3,862 questionnaires along with a valid informed consent form were returned (response rate of 12.11%). All questionnaires were checked for completeness and plausibility, and incomplete questionnaires were completed via telephone interviews. Nevertheless, 628 questionnaires had to be excluded from the present analysis due to a lack of several items or implausibility (n = 361, including n = 51 who did not meet the 100% ± 1% add up criterion for body representation in dream, as mentioned above), lack of the item assessing dream recall frequency (n = 43), multiple amputations, which made the interpretations of the statements inconclusive (n = 127), or congenital abnormalities in limb development, which is to be distinguished from an amputation (n = 97).

Thus, N = 3,234 amputees (18.46% women) who indicated their dream recall frequency were included in the analysis. The mean age was 64.37 years (SD = 15.89). A majority of 74.51% suffered from an amputation of the lower-limbs. On average, the participants had lost more than half of the respective limb (length of the residual limb = 41.83%, SD = 22.35). At the time of data acquisition, the amputation had been performed on average 33.06 years ago (SD = 22.74). Most of the participants (80.72%) stated that they used a prosthesis. During the preceding three months, the majority of participants had experienced some kind of post-amputation pain (PLP: 62.55%; RLP: 51.48%) as well as non-painful phantom sensations (56.91%). A telescopic distortion was indicated by 22.97% of the limb amputees. Less than one out of ten (9.14%) reported a history of a mental disorder. Seen individually, all of these items had missing values of < 1% each. These data served as regressors for the subsequent logistic regression analyses described below.

### Statistical analyses

First, we analyzed the dream recall frequency in our amputee sample and a representative German sample (N = 1,841, 993 women, mean age = 48.01 years, SD = 18.36; combined from [22] and [23]), by performing an ordinal logistic regression analysis including the regressors *group* (0 = representative sample, 1 = amputee sample), *sex* (0 = female, 1 = male), and *age*. We then performed another ordinal logistic regression on dream recall frequency in the amputee sample, including the site of and time since amputation, residual limb length, prosthesis use, life time prevalence of mental disorder, presence of post-amputation pain, and presence of non-painful phantom phenomena, controlling for age and sex.

Since we expected the assessed data on body representation in dreams being ordinal, and not necessarily normally distributed, we also performed ordinal logistic regression analyses for pooled percentage ranks (eleven ranks, Table 1), assuming a cumulative logistic model and including site of and time since amputation, residual limb length, prosthesis use, life time prevalence of mental disorders, presence of post-amputation pain, and presence of non-painful phantom phenomena (forced entry). We controlled for the effects of age, sex, and dream recall frequency. We performed a separate regression analysis for each category (impaired, intact, and not remembered body representation in dreams). However, due to relative redundancy of percentage statements, we adjusted the *p* values obtained in the three regression analyses applying Bonferroni correction. To check the equal slopes assumption [24], we used the score test and graphical techniques like plotting the empirical logits. Statistical analyses were carried out with SAS 9.2 (SAS, Cary, NC, USA).

**Table 1:** Percentage ranks of the body representation recall in dreams items (n = 2,156).

Rank	Body representation in dreams					
	Impaired		Intact		Not remembered	
	n	%	n	%	n	%
0_0%_	1265	58.67	630	29.22	831	38.54
1_1–10%_	309	14.33	124	5.75	152	7.05
2_11–20%_	158	7.33	100	4.64	158	7.33
3_21–30%_	98	4.55	98	4.55	91	4.22
4_31–40%_	59	2.74	84	3.90	70	3.25
5_41–50%_	90	4.17	175	8.12	136	6.31
6_51–60%_	38	1.76	74	3.43	38	1.76
7_61–70%_	29	1.35	83	3.85	42	1.95
8_71–80%_	35	1.62	140	6.49	67	3.11
9_81–90%_	11	0.51	83	3.85	71	3.29
10_91–100%_	64	2.97	565	26.21	500	23.19

## Results

### Dream recall frequency

The ordinal logistic regression analyses on dream recall frequency of amputees and members of a representative sample revealed significant influences of group membership and sex, with higher dream recall frequencies in amputees compared to members of the representative sample (standardized regression coefficient (SRC) = .237, χ^2^ = 210.1, *p* <. 001) and females compared to males (SRC = .080, χ^2^ = 29.1, *p* <. 001). Further, age was negatively associated with dream recall frequency (SRC = -.143, χ^2^ = 86.4, *p* <. 001). In total, these variables explained about 5% of the variation (R^2^ = .047) of dream recall frequency.

In the amputee sample, the majority of the participants (2,338, equal to 72.29%) were able to recall dreams and 1,311 participants (equal to 40.54%) indicated a dream recall at least once a week. Table 2 shows the dream recall frequencies in percent for each category for the entire amputee sample. Moreover, the regression analyses in the sample of amputees indicated that certain amputation-related sensations (presence of non-painful phantom sensations, a telescopic distortion, and residual limb pain) as well as a lifetime history of mental disorders were associated with higher dream recall frequency (see Table 3).

**Table 2:** Dream recall frequency for the entire sample (N = 3,234) and the sub sample with valid responses on the body representation in dream items (n = 2,156).

dream recall frequency	% of participants	
	entire sample	subsample
Never	27.71	---
Less than once a month	11.38	14.89
About once a month	8.53	11.83
Two or three times a month	11.84	16.84
About once a week	13.64	19.06
Several times a week	18.00	24.95
Almost every morning	8.91	12.43

**Table 3:** Results for ordinal logistic regression analyses of dream recall frequency (given validity of all other item values; n = 3,164).

Independent variables	Dream recall frequency	
	SRC	*χ*²
Age	-.138	41.5***
Sex+	.031	3.1
Site of amputation++	.038	3.3
Life time prevalence of mental disorders+++	.057	10.5**
Residual limb length	-.005	0.1
Elapsed time since amputation	.038	2.9
Prosthesis use+++	-.001	0.2
Presence of non-painful phantom sensations+++	.108	31.1***
Presence of a telescope+++	.047	6.3*
Presence of phantom limb pain+++	.002	1.0
Presence of residual limb pain+++	.049	7.8**
*R* ^2^	.053	

SRC = standardized regression coefficient

+0 = female, 1 = male; ++0 = upper-limb, 1 = lower-limb; +++0 = no, 1 = yes

* *p*<.05;

** *p*<.01;

*** *p*<.001

### Prevalence of body representation in dreams

The questionnaires of 2,156 participants (equal to 66.67% of the entire sample) contained valid data on body representation in dreams (averaged ratings for these items are depicted in Table 4). The distribution of dream recall frequency of this subsample is shown in Table 2. A minority of 484 participants (equal to 22.45%) of these subjects indicated that they were not able to remember their body representation in dreams (i.e., a rating of 100% for this category). Another 525 participants (equal to 24.35%) stated that they dreamed themselves as always intact, and only 61 participants (equal to 2.83%) dreamed their bodies as always impaired. The remaining amputees indicated a mix of at least two of the categories.

Seven-hundred-and-fifty-four participants (equal to 34.97%) recalled their bodies in recent dreams in a mixed fashion, meaning that in some dreams their body was intact and in others it was impaired. Even in this subgroup (n = 754), about one in two dreams (48.42%, SD = 27.49) contained an intact body representation. In a quarter of dreams, an impaired body representation could be recalled (27.24%, SD = 22.18), and roughly the same averaged percentage (24.33%, SD = 26.83) in this subgroup was indicated for dreams in which no body representation could be remembered.

**Table 4:** Percentage of estimates of the body representation in dreams (n = 2,156).

Category	Mean amount of dreams in % (standard deviation)
Impaired body representation	13.67 (24.53)
Intact body representation	47.72 (40.98)
Body representation not remembered	38.61 (41.37)

### Ordinal logistic regression on body representation in dreams

The ordinal logistic regression analyses (Table 5) explained 5.2 to 8.4% of the variation of recalled body representation in dreams. Older persons tended to report more dreams with intact body representation but less often dreams with impairments due to amputation and dreams without recall of body representation. Sex of participants was not associated with recalled body representation. As expected, participants who frequently recalled their dreams were more often able to remember their body representation in dreams (whether intact or impaired).

**Table 5:** Results for ordinal logistic regression analyses on body representation in dreams (given validity of all other item values; n = 2,112).

Independent variables	Body integrity in dreams					
	Impaired		Intact		Not remembered	
	SRC	*χ*²	SRC	*χ*²	SRC	*χ*²
Age	-.131	20.6***	.174	44.1***	-.148	30.7***
Sex+	-.036	2.1	.009	0.2	-.010	0.2
Dream recall frequency	.167	45.1***	.139	41.0***	-.184	68.8***
Site of amputation++	.094	10.3**	.006	0.1	-.047	3.4
Life time prevalence of psychiatric disorders+++	.045	3.9	-.004	0.0	-.003	0.0
Residual limb length	-.059	5.2	-.043	3.5	.068	8.7**
Elapsed time since amputation	-.005	0.0	-.112	17.2***	.105	14.3***
Prosthesis use+++	.004	0.0	.039	2.5	-.009	0.1
Presence of non-painful phantom sensations+++	.023	0.7	.011	0.2	-.006	0.1
Presence of a telescope+++	.050	4.0	.007	0.1	.009	0.2
Presence of phantom limb pain+++	.075	7.6*	.028	1.3	-.054	4.9
Presence of residual limb pain+++	.109	20.2***	-.044	4.0	.02	0.8
*R* ^2^	.084		.052		.068	

SRC = standardized regression coefficient

+0 = female, 1 = male; ++0 = upper-limb, 1 = lower-limb; +++0 = no, 1 = yes

* *p*<.01667 (.05 / 3);

** *p*<.00333 (.01 / 3);

*** *p*<.00033 (.001 / 3)

*p* values Bonferroni corrected for multiple testing across the regression analyses

A lower-limb amputation significantly predicted an impaired body representation recall. The elapsed time since the amputation was negatively correlated with the percentage of dreams with an intact body representation, i.e., the longer the time interval between the amputation and study participation, the fewer dreams with intact body representation were reported. The elapsed time since amputation was also related to higher indications of dreams without any recall of body representation in the dreams. Residual limb length and impaired or intact body representation recall tended to be negatively associated, but the only significant association found was with not remembering the body in dreams, i.e., the longer the residual limb, the higher the percentage of dreams in which no body representation could be recalled. Prosthesis use, a history of mental disorder, or the presence of non-painful phantom sensations, including the presence of a telescopic distortion, were not significantly correlated with recalled body representation in dreams.

Finally, post-amputation pain had a significant association with recalled body representation. The presence of both PLP and RLP predicted individually and specifically the recall of an impaired body representation in dreams. There were no such significant associations with the other body representation recall categories.

## Discussion

The present study investigated body-related dream content in a large number of limb amputees using an epidemiological approach. Although only a minority of dreams contained bodily impairments, we demonstrated that there is a positive relationship between post-amputation pain in the waking state and the recall of an impaired body representation in dreams, which might give an insight into the interaction of physical and mental body representations, mediated by aversive somatosensory experiences.

In contrast to previous studies (e.g., [16]), we used an item assessing the percentage of intact, impaired and not remembered body representation, probably yielding more inter-individual differences in body-related dream content recall. About one quarter of limb amputees recalled their body representation in dreams as always intact, with less than 3% dreaming themselves as always impaired, which is in line with results of previous studies [16], [25], [26]. Mulder et al. [16] discussed this finding in the light of a potential, genetically mediated body model, which mostly remains unaffected from the adaptation processes occurring after amputation. Additionally, two recent studies found that congenital paraplegics mainly recalled dreams in which their body was not disabled [27], [28]. Voss et al. [28] interpreted their results as supporting the idea that dream content draws information from innate body representations, a concept which was introduced by Melzack et al. [29]. The missing influence of the length of the residual limb as well as a restitution of body integrity through prosthesis use on the recalled body representation in the present study seemingly supports this hypothesis. However, the high proportion of more than one third of limb amputees who dreamed themselves as both intact and impaired suggests that there are other influencing factors.

Previous studies indicated a certain flexibility of body representation in dreams after changes in physical conditions. Newton [30] asked paralyzed participants to keep a dream diary, and its analysis revealed that recently paralyzed people reported more kinesthetic dream contents than long-termed paralyzed participants. The results of the present study indicate a similar effect: the longer the amputation dated back, the fewer dreams with intact body could be remembered, highlighting that dreaming is not solely influenced by the recent past, but reverts to experiences made in the entire life [31]. This indicates that both innate factors and life experiences may affect the body representation in dreams. The effect of life experiences is reflected in the finding that age is negatively related to impaired body representation and positively related to an intact body representation in dreams, a finding previously reported by Mulder et al. [16]. The negative correlations between not remembering one’s body in dreams and age as well as time since amputation, however, might only emphasize the generally reduced recall of dream content characteristics at an older age [22]. Another possible interpretation of this result is that the longer time spent at sleeping/dreaming the more the amputees might benefit from the emotional modulation effect related to sleep and dreams [32] and thus the less the amputees need to dream of the own body.

From a methodological viewpoint, the present results indicate that dream recall frequency should be considered as a possible confounding variable for the percentage of body representation in dreams. First, we showed that members of the amputee group had higher dream recall frequencies compared to a representative German control sample; this might be explained by the low response rate in this study, perhaps related to the fact that the respondents might be rather open to express their psychological state and, thus, recall their dreams with a higher frequency (cf. [33]). Prospectively, the comparison between body-related dream recall frequency in amputees (61.39% in the present sample) and healthy controls might yield interesting add-on information. Further, the differences between amputees and healthy controls might be related to the high prevalence of disturbed sleep in amputees [1], which has been shown to be positively associated with dream recall frequency [34]. However, amputation-related experiences—except for PLP—were highly correlated with dream recall frequency, with the strongest effect for the presence of non-painful phantom sensations. This might be explained by inter-individual sensitivity to inner processes, since a study investigating the effect of acquiring autogenic training skills showed that the associated increased sensitivity to inner processes increases dream recall frequency [35].

Additionally, we found a significant positive association between dream recall frequency and the percentage of impaired or intact body representation in dreams. Whereas previous studies excluded subjects with low or no dream recall [16], the present findings indicate that the ability to recall body-related dream content is enhanced in subjects with higher dream recall frequency. This confirms previous findings suggesting that dream content estimates assessed by questionnaires are only weakly correlated with data obtained in diary studies, especially in subjects who seldom recall their dreams [17]. Thus, entering dream recall frequency into the regression analysis of dream content measures can be seen as a methodological improvement compared to previous studies on recalled body representation in limb amputees.

We found a significant effect of amputation site on remembered body representation in dreams, i.e., lower-limb amputations were associated with higher frequency of dreams with an impaired body than upper-limb amputations. This contradicts the finding of Mulder et al. [16]. These authors also compared arm and leg amputees in order to explore possible differences on body-related dream content and found no such effect. The majority of amputations, however, is performed on lower limbs (in Germany, for instance, 71.92% of amputees live with the loss of one or both legs [36]), leading to an asymmetric distribution of amputation site. As a consequence, Mulder et al. [16] compared six upper-limb amputees with 69 lower-limb amputees, achieving only low statistical power. In the present study, this problem was accounted for by the large number of participants. The higher recall of an impaired body representation associated with leg amputations might be due to the higher quality of health-related life in upper-limb amputees [37] and potentially higher risk of lower-limb amputees to develop severe PLP [38]. Further, recent evidence suggests that leg movements in dreams are more frequently recalled than arm movements [27], which might highlight the importance of mobility in daily life.

Our major finding is the association of painful post-amputation experiences in the waking state and remembered body-related dream content: the presence of post-amputation pain, i.e., both PLP and RLP, was associated with higher recall of an impaired body representation. This is an interesting finding, since the general presence of non-painful phantom sensations had no significant association, indicating that the mere presence of a phantom limb is not sufficient for dreaming of oneself as intact. This clearly indicates that there might be other factors affecting the body representation in dreams than the proposed innate body model [16]. These findings rather support the assumption that there is a relationship between the salience of a waking experience and the probability to dream about it [18]. In case of limb amputation, post-amputation pain might remind the amputee of the physical impairments and its negative consequences which are reflected in a more frequent recall of an impaired body. Amputees suffering from PLP report lower levels of quality of life [39] and this emotional distress associated with the experience of post-amputation pain might determine higher frequencies of negative dream content (i.e., an impaired body representation). Remarkably, this association was exclusively found for painful post-amputation experiences. The presence of non-painful phantom experiences or of a telescopic distortion of the phantom—which might likewise draw the amputee’s attention on his or her impairments during daily life—did not influence the frequency of impaired body representation in dreams. Thus, the specific associations to post-amputation pain reported here suggest an influence of suffering rather than physical alterations. To further explore this hypothesis, it would be useful to explicitly measure the amount of distress caused by the amputation and/or affective acceptation of limb loss.

Alternatively, the presence of post-amputation pain might change the focus of retrospection for dreams, improving the recall of amputation-related content. Since the intensity of dreamed emotions is positively correlated with dream recall frequency [40], the recall of probably negatively toned amputation-related dreams might be facilitated. Further, amputees with post-amputation pain reported more disturbed sleep and awakening during nights than pain-free amputees [1], which might cause a rather elaborated memory of body-related dream content in this subgroup. Prospective studies should include measures of sleep and dream quality, including the frequency of nightmares, to validate this hypothesis. However, these alternatives appear to be rather unlikely since the presence of post-amputation pain did not affect dream recall frequency per se.

Furthermore, post-amputation pain was found to be a risk factor for the prevalence of depressive symptoms, from which more than 25% of amputees are suffering [41]. Cognitive processing in depression is characterized by a bias in recalling negative self-referred information [42], [43], [44]. However, the percentage of participants who reported mental disorders in the present study was lower than expected based on amputee data reported in the literature [41], [45], [46]. Further research is necessary to replicate our finding that the presence of a mental disorder does not affect the percentage of an impaired body representation in dreams, as one might expect that patients with certain mental disorders are more likely to ruminate about their impairment in waking and, thus, report more dreams with bodily impairments.

Further, the survey itself might induce a cognitive bias on recalled dream content. Previous cognitive tasks can affect the recall of emotional memory [47]. In the present study, amputees suffering from current post-amputation pain had to consider in detail their situation during the course of the questionnaire; the dream-related questions, however, were located at the end of the survey. This order might induce a recall bias for an impaired body in amputees suffering from post-amputation pain. In order to validate the present findings it could be useful to apply other dream collection methods. Alessandria et al. [48] and Vetrugno et al. [49] assessed dream content in amputees after awakening from REM sleep and asked them for their dreamed body representation. However, Alessandria et al. [48], for instance, found that only three of six amputees were able to recall body-related dream content instantly after awakening (all three amputees reported an intact body representation during the dream). Due to the small sample size in these sleep laboratory studies, generalization of the results is limited. Another option to collect dream reports from larger samples is the diary approach [17], [31], minimizing the bias due to retrospectively eliciting dream characteristics.

## Conclusion

In the present study we found significant associations between post-amputation pain in the waking state and recalled body-related dream content in a large sample of limb amputees. The findings indicate that negatively toned experiences in the waking state may interact with body-related dream content. Thus, body representation in dreams cannot simply be explained by an innate body model relatively insensitive to physical changes. Thus, the results support the continuity hypothesis of dreaming [14]. Even though our results are conclusive, the identification of influencing factors is complex. The relatively low amount of explained variance in our regression models emphasizes the importance of other psychological and physiological factors. To identify such influencing factors, an interesting follow-up of our study would be to assess body representations in dreams in a large sample of subjects who are congenitally disabled. Previous studies on dream content relying on sensory experiences, which had never occurred in life [50], suggest that even subjects who never experienced an intact body can dream about it because it is highly prevalent in their daily life. Studying the dream content in congenital amputees might help to understand the psychological challenges of this group.
